# Apple polysaccharide improves age-matched cognitive impairment and intestinal aging through microbiota-gut-brain axis

**DOI:** 10.1038/s41598-024-67132-4

**Published:** 2024-07-13

**Authors:** Wenming Zhang, Yuchun Zhong, Zhuoya Wang, Furui Tang, Cihua Zheng

**Affiliations:** 1 Department of Hepatobiliary Surgery, The Second Affiliated Hospital of Nanchang University, 330000, Nanchang, Jiangxi People’s Republic of China; 2 Department of Rehabilitation Medicine, The Second Affiliated Hospital of Nanchang University, Nanchang, Jiangxi People’s Republic of China; 3 The Institute of Translational Medicine, the Second Affiliated Hospital of Nanchang University, Nanchang University, 1 Minde Road, Nanchang, 330006 Jiangxi People’s Republic of China

**Keywords:** Aging, Apple polysaccharides, Cognitive disorder, Intestinal mucosal barrier, Gut microbiota, Microbiology, Cognitive ageing, Intestinal diseases

## Abstract

The Apple polysaccharides (AP), extracted from the fruit of apple, has been used to treat multiple pathological diseases. In this study, we evaluated the effects of AP on cognitive impairment and intestinal aging in naturally aging mice. As a result, it was found that AP could improve spatial learning and memory impairment in aging mice through the Morris water maze experiment. Additionally, AP intervention can upregulate the expression of nerve growth factor (BDNF), postsynaptic marker (PSD95), and presynaptic marker (SYP) proteins. Moreover, AP can enhance total antioxidant capacity, reduce the level of pro-inflammatory cytokine, and inhibit the activation of the NF-κB signaling pathway, exerting anti-inflammatory and antioxidant functions. And the administration of AP restored intestinal mucosal barrier function, reduced the expression of aging and apoptosis related proteins. The administration of AP also altered the gut microbiota of mice. At the genus level, AP decreased the abundance of *Helicobacter* and *Bilophila*, while increased the abundance of *Lactobacillus* and *Bacteroides*. In summary, these data demonstrate that AP treatment can alleviate cognitive impairment, oxidative stress, and inflammatory reactions, repair the intestinal mucosal barrier, reduce intestinal aging, and alter specific microbial characteristics, ultimately improving the health of the elderly.

## Introduction

Aging is a spontaneous and inevitable process in which the body's physiological and psychological adaptability to the environment gradually decreases and tends towards death^1^. The results of China's seventh population census show that China's aging population is further deepening, and the world's aging population is also increasing^2^. Population aging brings heavy pressure to the healthcare system. Aging is often accompanied by low immune function, skin relaxation, decline in memory and cognitive ability, metabolic disorder, and decline in stem cell differentiation, and is related to a variety of diseases, such as cancer, diabetes, autoimmune diseases. Aging is accompanied by the failure of various organ functions throughout the body^3^. Due to its immune and nutrient intake functions, the intestine is considered an important organ that regulates the body's ability to extend lifespan^4^. The father of probiotics, *Mechinikov*, said, " Aging begins with the intestines. Only when the intestines are healthy can the body be healthy. The toxins produced by the gut microbiota are the main cause of aging and disease in the body."^5^. The gut microbiota present in the intestine will experience dysbiosis with aging, resulting in a decrease in beneficial bacteria and metabolites, an increase in intestinal lipopolysaccharide and inflammation levels, damage to intestinal barrier function, and accelerated aging process^6^.

The gut microbiota refers to all microbial colonies that are designated to grow in the host's intestines, known as "hidden organs"^7^. Under normal circumstances, there are approximately 100 trillion bacteria in the adult human body, of which 80% exist in the intestines^8^. The gut microbiota contains approximately 3.3 million genes, which is 150 times the number of genes in humans^8^. Among them, *Firmicutes* and *Bacteroidetes* are the most important phyla, representing the bacterial core of the human microbial community^9^. Research has shown that as age increases, the community structure and function of gut microbiota change, including changes in microbial diversity and relative abundance^10^. In addition, the level of lipopolysaccharides (LPS) in the intestinal cavity increases, and the abundance of short chain fatty acids with anti-inflammatory effects decreases, inducing inflammatory reactions and accelerating intestinal aging^11^. As a cell wall immune stimulatory component of Gram-negative bacteria, LPS can bind to toll like receptor 4 (TLR4) in their lipid A region, forming a complex that activates the nuclear factor-κB (NF-κB) signaling pathway, leading to the synthesis and secretion of inflammatory factor, which destroy the permeability of mucosa, reduce tight junction proteins and induce cell aging^12^. However, a study suggests that altering the composition of gut microbiota and reversing probiotic levels can regulate barriers and counteract intestinal aging^13^. Therefore, it is a way to maintain intestinal homeostasis and delay intestinal aging by regulating the gut microbiota, improving intestinal mucosal barrier and reducing the level of pro-inflammatory cytokines^13^.

Although aging is an irreversible and inevitable process, the speed of aging is controllable, and traditional Chinese medicine molecules have unique theories and rich experience in delaying aging^14^. Epidemiological studies have shown that eating apples can reduce the risk of chronic diseases such as cancer, cardiovascular disease, asthma and diabetes^15^. In addition, basic research has shown that apple polysaccharides (AP) can reduce the risk of cardiovascular disease, cancer, and neurodegenerative diseases^16^. The results of in vitro and in vivo experimental studies indicate that AP can prevent the occurrence of chemically induced chronic colon cancer in mice and inhibit the migration and invasion of colorectal cancer cells ^17^. The gut microbiota of mammals largely relies on dietary polysaccharides as an energy source, and most polysaccharides also require the participation of gut microbiota in degradation^18^. The dietary polysaccharides that reach the colon have a significant impact on the ecology and balance of gut microbiota^19^. Research has shown that AP can regulate the composition of gut microbiota, alter the concentration of short chain fatty acids in the intestinal lumen, improve chronic inflammation, and reduce intestinal permeability in high-fat induced mice^20^. However, there are currently no reports on the use of AP to delay intestinal aging. In this study, we evaluated the composition of the microbiota, intestinal permeability, and inflammatory response to detect the regulatory effect of AP on intestinal aging in 18 months mice, and further explored potential mechanisms.

## Results

### AP improved memory deficit and neuronal damage in aging mice

**Figure 1:**
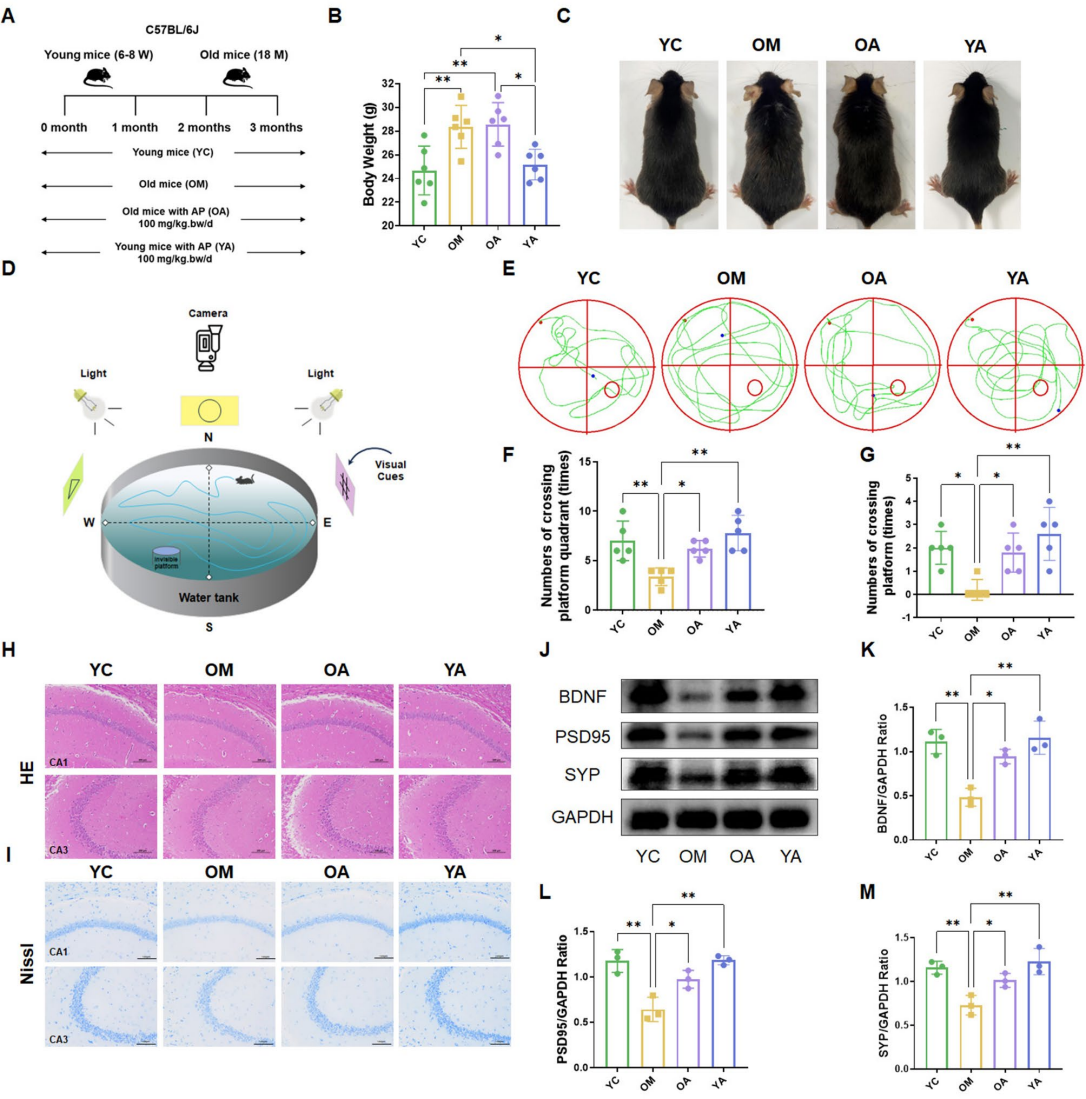
AP improved memory deficit and neuronal damage in aging mice. (**A**) Experimental design. (**B**) Mouse body weight. (**C**) General status. (**D-G**) Morris water maze test. (**H**) Morphology of mouse hippocampus tissues stained with H&E. (**I**) Representative micrographs of hippocampal CA1 and CA3 regions were analyzed by Nissl staining. (**J-M**) Expression of BDNF, PSD95 and SYP in brain tissue of mice, original blots were presented in Supplementary Figures Fig. S1. YC, young mice group; OM, aging mice group; OA, aging mice with 100 mg/kg AP group; YA, young mice with 100 mg/kg AP group. Scale bar, 100 μm. Data are presented as the mean ± SD. *P < 0.05; **P < 0.01.

To verify the effect of AP on delaying aging, this study treated mice with AP for 3 months (Fig. 1A). As shown in Fig. 1B, we observed a significant difference in weight between young and elderly mice, and AP had no significant effect on weight. However, the administration of AP improved mouse hair, which was smoother and thicker (Fig. 1C). Furthermore, the memory function of mice was evaluated, and it was found that the treatment of AP obviously improved the memory function of mice, which showed that the number of cross-platform quadrants and cross-platform times significantly increased (Fig. 1D-G). Next, we used HE staining to evaluate the effect of AP on the pathological changes of brain tissue in aging mice (Fig. 1H). In the control group (YC), the HE staining morphology of neurons was normal, showing around nucleoli or oval shape, with clear nucleoli and regular arrangement. On the contrary, the aging group (OM) showed neuronal changes, such as dark black nucleus and decreased cytoplasm, and AP could alleviate this pathological change (OA). Nissl staining in hippocampus showed that compared with OM mice, the number of neurons in hippocampus of aging mice treated with AP obviously increased, and the administration of AP preserved the survival of neurons in hippocampus of aging mice (Fig. 1I). Similarly, compared with the control group (YC), the administration of AP in young mice (YA) has been improved accordingly. To further clarify the ability of AP to improve learning and memory, we detected the protein expressions of brain-derived neurotrophic factor (BDNF), postsynaptic density 95 (PSD95) and synaptophysin (SYP). As shown in Fig. 1J–M, the expression levels of BDNF, PSD95 and SYP in hippocampus of aged mice were decreased, while they were up-regulated after AP treatment. The above results indicated that AP can obviously improve the degeneration of the nervous system in aging mice.

### AP improved inflammatory factors and antioxidant stress of aging mice

**Figure 2:**
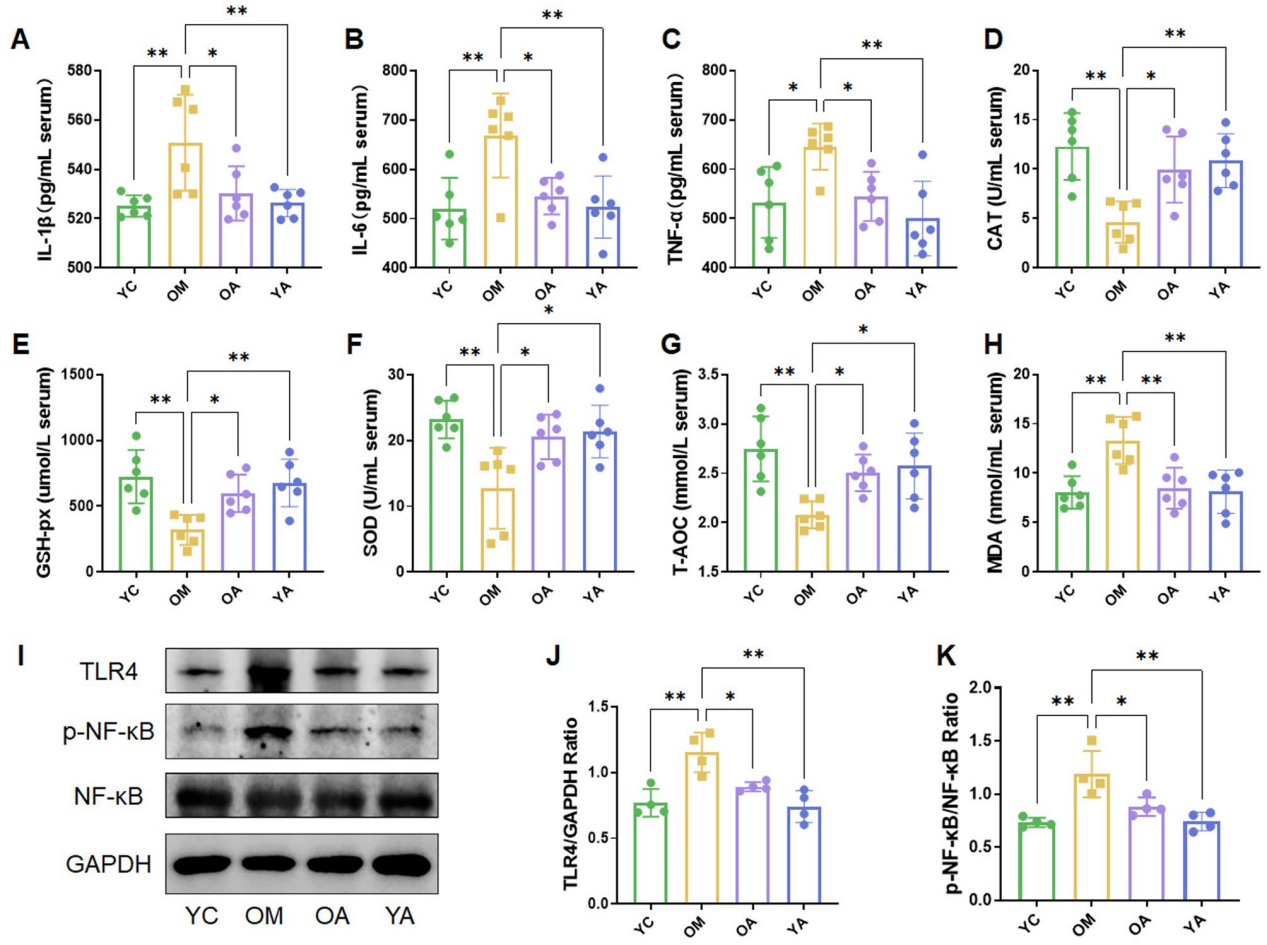
AP improved anti-inflammatory factors and antioxidant stress of aging mice. (**A-C**) Serum proinflammatory cytokines IL-1β, IL-6 and TNF-α levels. (**D-H**) Serum catalase (CAT), glutathione peroxidase (GSH-px), superoxide dismutase (SOD), total antioxidant capacity (T-AOC) and malondialdehyde (MDA) activity levels. (**I-K**) Expression of key proteins in TLR4/NF-κB signaling pathway, original blots were presented in Supplementary Figures Fig. S2. YC, young mice group; OM, aging mice group; OA, aging mice with 100 mg/kg AP group; YA, young mice with 100 mg/kg AP group. Data are presented as the mean ± SD. *P < 0.05; **P < 0.01.

Aging is closely related to inflammation and oxidative stress levels in the body. Therefore, we evaluated the effects of AP on inflammation and oxidative stress in mice. The results showed that the administration of AP significantly reduced the levels of IL-1β, IL-6 and TNF-α in serum compared to the OM group (Fig. 2A–C, *P* < 0.05). In addition, compared with the OM group, the serum levels of CAT, GSH-px, SOD and T-AOC were sharply increased in OA group (Fig. 2D–G, *P* < 0.05). Meanwhile, compared with the OM group, the serum MDA level was obviously reduced in OA group (Fig. 2H, *P* < 0.01). Furthermore, we detected the expression of key proteins in the classic inflammatory signaling pathway TLR4/NF-κB in colon tissue. The results showed that compared with the YC group, the TLR4 protein level in the OM group mice was sharply upregulated, accompanied by an increase in NF-κB phosphorylation level (p-NF-κB), while the administration of AP significantly inhibited the activation of the signaling pathway (Fig. 2I–K, *P* < 0.05). Therefore, AP can reduce the level of inflammation in aging mice and improve their antioxidant capacity.

### AP has a protective effect on the intestinal barrier in aging mice

**Figure 3:**
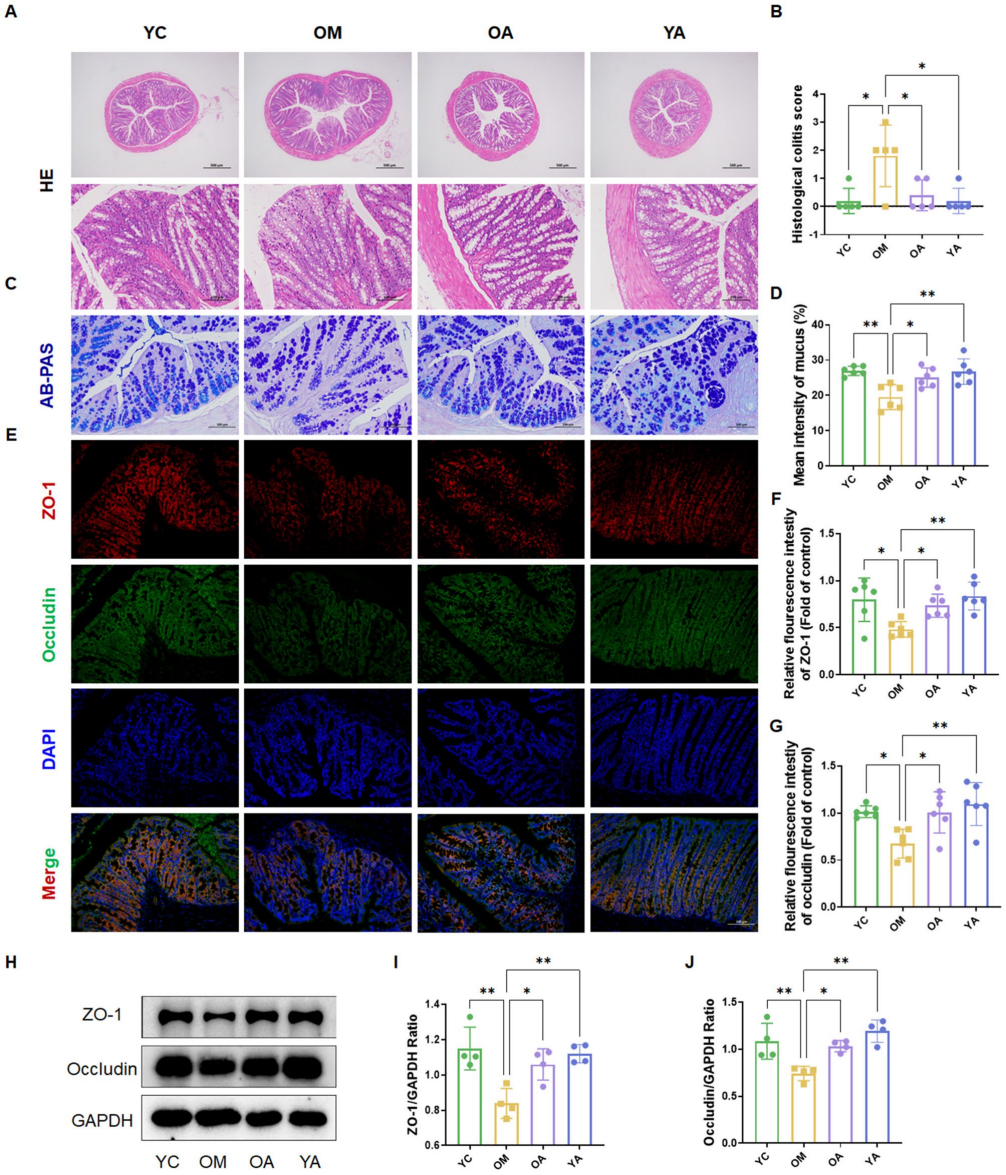
AP has a protective effect on the intestinal barrier in aging mice. (**A, B**) H&E staining and pathological score of colon tissue. (**C, D**) Representative images and quantitative analysis of AB/PAS staining in colon tissue. (**E–G**) Representative immunofluorescence images and quantitative analysis of tight junction proteins (ZO-1, Occludin) in colon sections. (**H-J**) Expression of tight junction protein (ZO-1, Occludin) in colon tissue of mice, original blots were presented in Supplementary Figures Fig. S3. YC, young mice group; OM, aging mice group; OA, aging mice with 100 mg/kg AP group; YA, young mice with 100 mg/kg AP group. Scale bar, 100 μm. Data are presented as the mean ± SD. *P < 0.05; **P < 0.01.

The intestinal mucosal barrier function plays an important role in aging. Next, we further evaluated the impact of AP on the colon morphology and intestinal barrier function. As shown in Fig. 3A and B, the colon structure of aging mice was disrupted, and inflammatory cells infiltrated, and AP effectively improved this situation. The AB/PAS staining results showed that aging led to a decrease in the number of goblet cells and thinning of the mucosal layer in colon tissue, and the administration of AP significantly reversed this phenomenon (Fig. 3C and D). Tight junction (TJ) proteins play a crucial role in controlling intestinal permeability. Subsequently, we used immunofluorescence staining (Fig. 3E–G) and western blotting (Fig. 3H–J) to detect the protein expression of tight junction proteins ZO-1 and occludin. The results showed that compared with the YC group, the expression of TJ proteins in the intestine of aging mice was significantly reduced (*P* < 0.05), while the administration of AP improved intestinal barrier function (*P* < 0.05). These results revealed that AP can improve the disruption of intestinal TJ barrier caused by aging.

### AP delayed intestinal aging in mice

**Figure 4:**
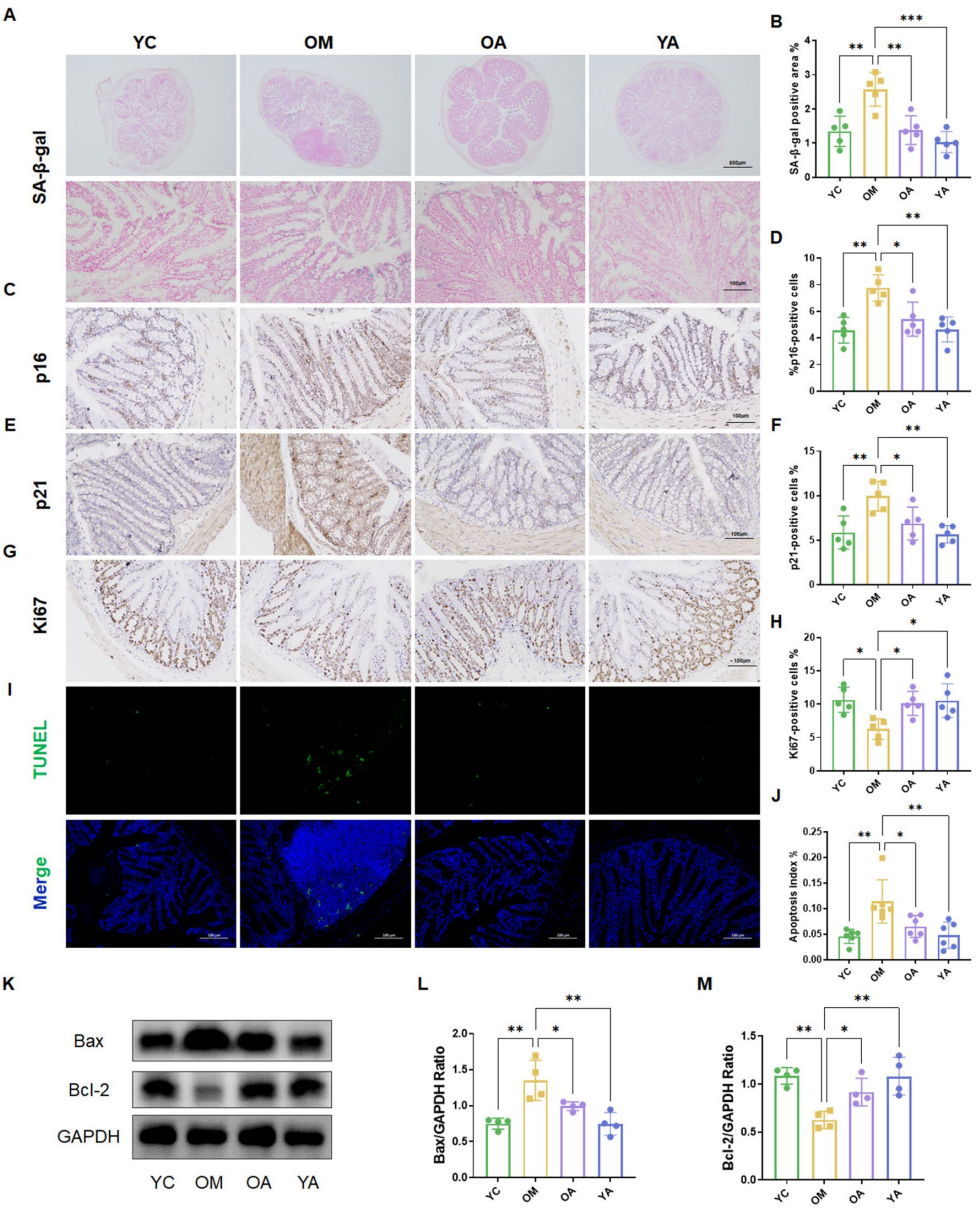
AP delayed intestinal aging in mice. (**A, B**) Representative images and quantitative analysis of SA-β-gal staining in colon tissue. (**C-F**) Representative images and quantitative analysis of p16 and p21 immunohistochemical staining in colon tissue. (**G, H**) Representative images and quantitative analysis of Ki67 immunohistochemical staining in colon tissue. (**I, J**) Representative fluorescent images and quantitative analysis of TUNEL staining in colon sections. (**K-M**) Expression of apoptosis related proteins (Bax, Bcl-2) in mouse colon tissue, original blots were presented in Supplementary Figures Fig. S4. YC, young mice group; OM, aging mice group; OA, aging mice with 100 mg/kg AP group; YA, young mice with 100 mg/kg AP group. Scale bar, 500 μm or 100 μm. Data are presented as the mean ± SD. *P < 0.05; **P < 0.01; ***P < 0.001.

Further, we observed whether AP has the effect of delaying intestinal aging, and we evaluated the expression level of tissue aging markers and cell apoptosis. Firstly, we conducted SA-β-gal staining analysis on colon tissue and found that the positive area of SA-β-gal in colon tissue of aging mice increased compared to control mice (YC, *P* < 0.01), and the administration of AP significantly reduced the positive area of SA-β-gal (Fig. 4A,B). Immunohistochemical results showed that compared with YC and YA mice, the levels of p16^lnk4a^ (p16) and p21^waf1^ (p21) proteins were elevated in the colon tissue of aging mice (OM group), and the administration of AP significantly downregulated the levels of aging marker proteins (Fig. 4C–F). Additionally, we observed a decrease in the number of Ki67^+^ progenitor cells in old mice compared to young mice, which were rescued by AP treatment (Fig. 4G,H). Moreover, we elucidated the effect of AP on intestinal cell apoptosis by using TUNEL staining and western blotting. The results of TUNEL staining showed that compared with the YC group, the number of TUNEL positive cells in the colon tissue of aging mice significantly increased, and the administration of AP reversed this result (Fig. 4I,J). As shown in Fig. 4K–M, the level of Bax increased and Bcl-2 decreased in OM group (*P* < 0.01), while AP sharply decreased the expression of Bax and obviously increased the level of Bcl-2 (*P* < 0.05). These results indicated that AP can reduce intestinal tissue cell apoptosis and delay intestinal aging in mice.

### AP altered the composition of gut microbiota in aging mice

**Figure 5:**
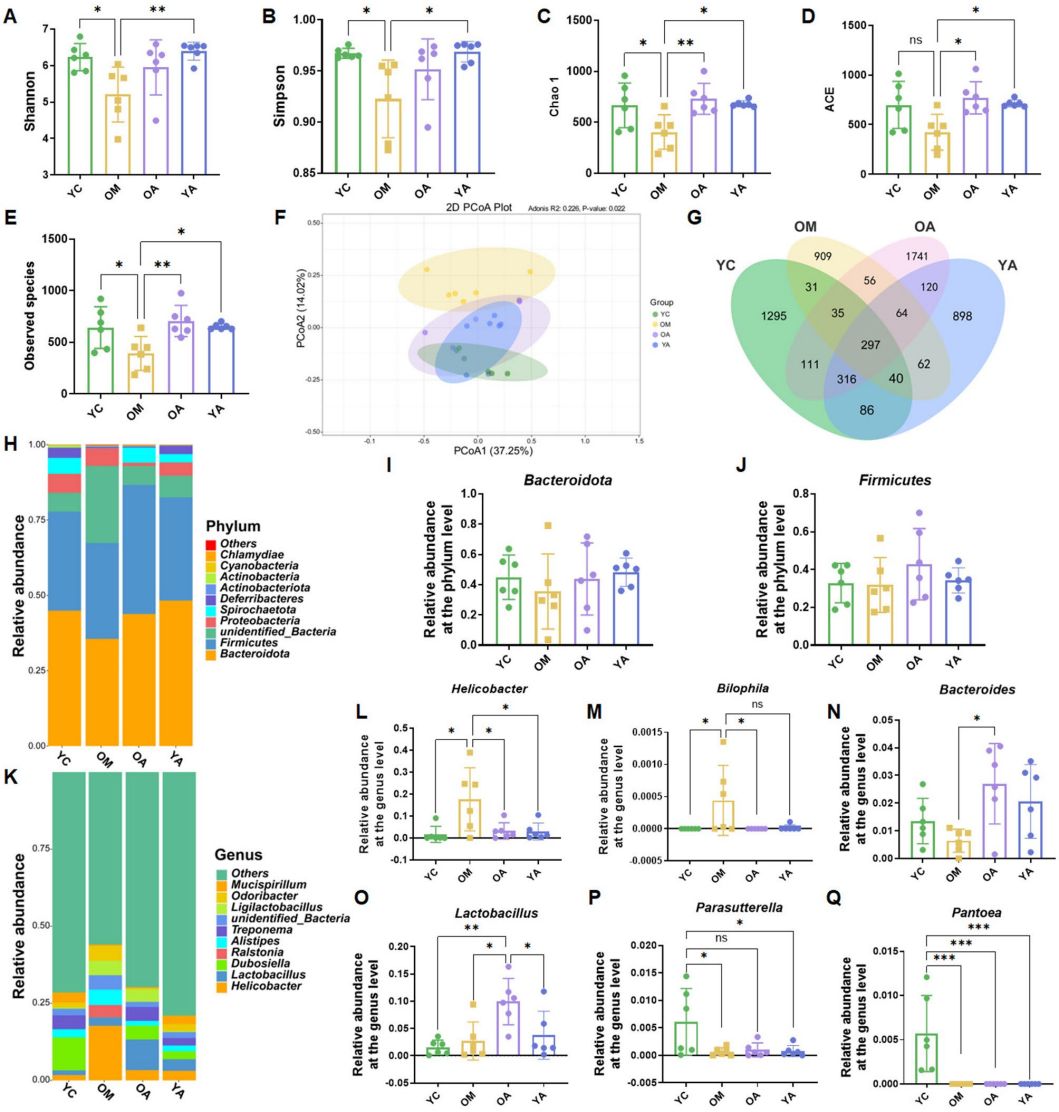
AP altered the composition of gut microbiota in aging mice at the phylum and genus levels. (**A**) Shannon index. (**B**) Simpson index. (**C**) Chao 1 index. (**D**) ACE index. (**E**) Observed species. (**F**) PCoA of the β diversity index. (G) Venn diagram. (**H**) Relative abundance of the identified fecal microbiota at the phylum level. (**I**) *Bacteroidetes*. (**J**) *Firmicutes*. (**K**) Relative abundance of the identified fecal microbiota at the genus level. (**L**) *Helicobacter*. (**M**) *Bilophila*. (**N**) *Bacteroides*. (**O**) *Lactobacillus*. (**P**) *Parasutterella*. (**Q**) *Pantoea*. YC, young mice group; OM, aging mice group; OA, aging mice with 100 mg/kg AP group; YA, young mice with 100 mg/kg AP group. Data are presented as the mean ± SD. ns, *P* > 0.05; *P < 0.05; **P < 0.01; ***P < 0.001.

16S rDNA gene sequencing was used to analyze the fecal microbiota of mice in each group. The results showed that aging mice α-diversity was significantly reduced, and administration of AP promoted its recovery, including Shannon (Fig. 5A), Simpson (Fig. 5B), Chao1 (Fig. 5C), ACE (Fig. 5D), and observed species (Fig. 5E). Principal coordinate analysis (PCoA) confirmed that aging (OM) caused the separation of gut microbiota from the young group (YC) of mice, while AP administration led to the OA and YA groups approaching each other and tending towards the YC group (Fig. 3F). In addition, the Venn diagram showed a total of 297 OTUs between the 4 groups, with 1295, 909, 1741 and 898 unique OTUs in the YC, OM, OA, and YA groups, respectively (Fig. 5G). There are 2211, 1494, 2740, and 1883 OTUs in the YC, OM, OA, and YA groups, with the percentages of common OTUs in each group being 13.43% (297/2211), 19.87% (297/1494), 10.83% (297/2740), and 15.77% (297/1883), respectively (Fig. 5G).

**Figure 6:**
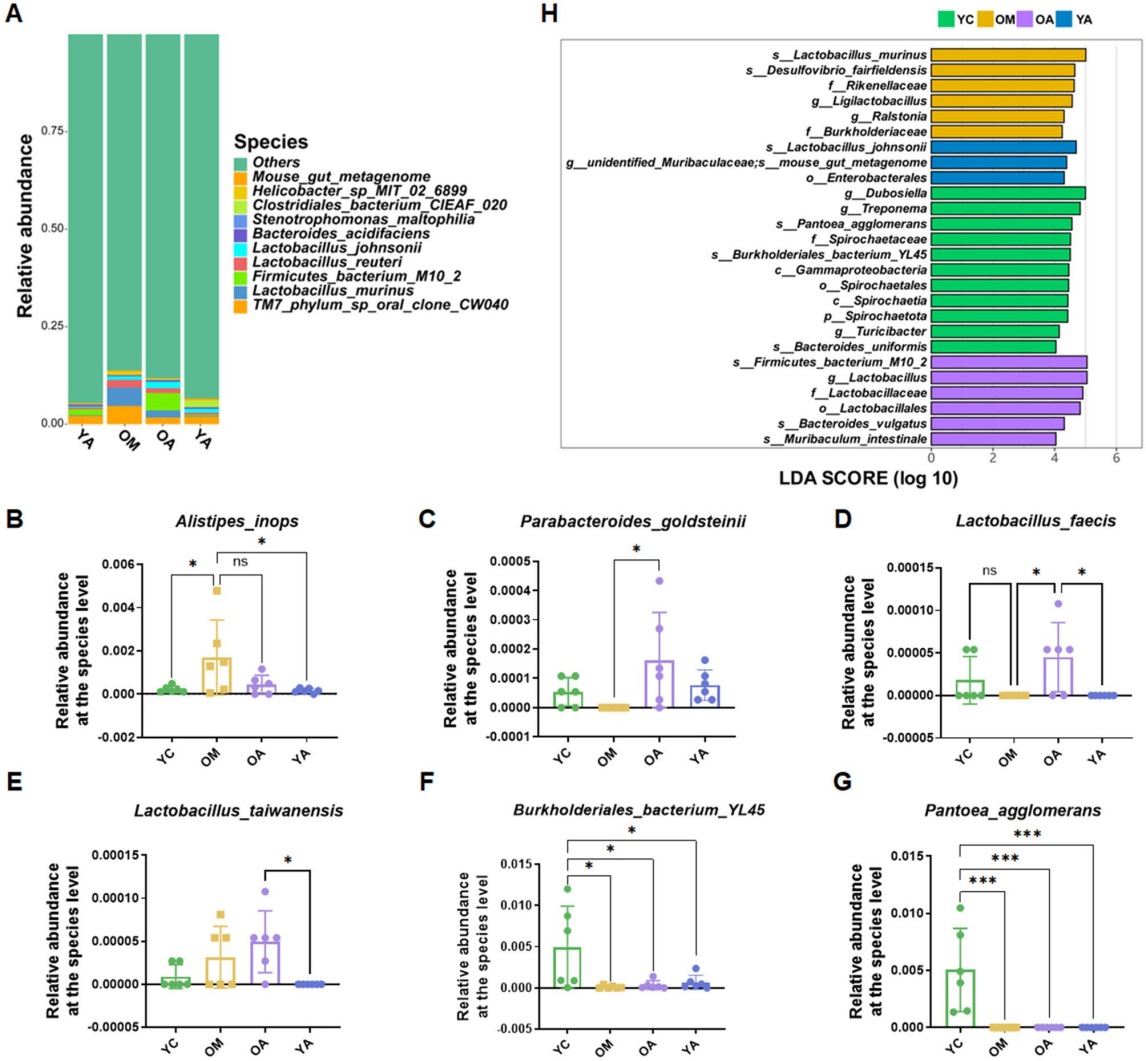
AP altered the composition of gut microbiota in aging mice at the species level. (**A**) Relative abundance of the identified fecal microbiota at the species level. (**B**) *Alistipes inops*. (**C**) *Parabacteroides goldsteinii*. (**D**) *Lactobacillus faecis*. (**E**) *Lactobacillus taiwanensis*. (**F**) *Burkholderiales bacterium YL45*. (**G**) *Pantoea agglomerants*. (**H**) LEfSe analysis on fecal microbiome of the mice. YC, young mice group; OM, aging mice group; OA, aging mice with 100 mg/kg AP group; YA, young mice with 100 mg/kg AP group. Data are presented as the mean ± SD. ns, *P* > 0.05; *P < 0.05; ***P < 0.001.

Next, we further explored the composition of gut microbiota and gut disease-related bacteria at the phylum, genus, and species levels. At the phylum level, *Bacteroidetes* and *Firmicutes* were dominant bacteria in the fecal microbiota, and there was no significant difference among the groups (Fig. 5H–J). At the genus level, *Helicobacter* and *Lactobacillus* were the dominant genera of fecal microbiota (Fig. 5K). Further analysis showed that the abundance of *Helicobacter* and *Bilophila* in aging mice significantly increased, while the administration of AP significantly reduced the relative abundance of these bacterium (Fig. 5L,M). Meanwhile, we also observed that the administration of AP sharply increased the relative abundance of *Lactobacillus* and *Bacteroidetes* (Fig. 5N,O). In addition, we also found that the young group of mice (YC) who were not treated with AP had higher relative abundance of *Parasutterella* and *Pantoea* in fecal microbiota (Fig. 5P,Q). Subsequently, we analyzed the differential bacteria at the species level (Fig. 6A). The species level analysis results showed that there was a high abundance of *Alistipes_inops* in the fecal microbiota of aging mice. After administration of AP to aging mice, the relative abundance of *Alistipes_inops* decreased, but there was no significant difference compared to the OM group (Fig. 6B). Further analysis revealed that after administration of AP to aging mice (OA), the relative abundance of *Paraacteroides goldsteinii* and *Lactobacillus faecis* in fecal microbiota notably increased compared to the OM group (Fig. 6C,D). Meanwhile, the relative abundance of *Lactobacillus taiwanensis* in the fecal microbiota of OA group mice increased (Fig. 6E). In addition, we also observed high relative abundances of *Burkholderiales'bacterium_YL45* and *Pantoea-agglomerants* in the fecal microbiota of young mice without AP administration (YC) (Fig. 6F,G). The linear discriminant analysis effect size (LEfSe) analysis demonstrated that *Lactobacillus_murinus* (at species), *Desulfovibrio_fairfieldensis* (at species), *Rikenellaceae* (at phylum), *Ligilactobacillus* (at genus), *Ralstonia* (at genus), *Burkholderiaceae* (at family) were enriched in the OM group, *Firmicutes_bacterium_M10_2* (at species), *Lactobacillus* (at genus), *Lactobacillaceae* (at genus), *Lactobacillales* (at order), *Bacteroides_vulgatus* (at species), *Muribaculum_intestinale* (at species) enriched in the OA group (Fig. 6H).

## Discussion

In this study, the therapeutic effect of AP on delaying intestinal aging in mice was evaluated by administering it to mice that grew naturally to 18 months. We explored the levels of inflammation and oxidative stress, intestinal aging markers, intestinal barrier function and fecal microbial composition in mice, and confirmed that AP can delay intestinal aging.

Neuronal degeneration is the main pathological process in aging, which can lead to progressive memory loss and cognitive impairment. Previous studies have reported that aging mice exhibit statistically increased oxidative damage and poorer cognitive function in the Y-maze test^21^. Similarly, D-galactose induced simulated aging mice can exhibit neuronal damage accompanied by cognitive decline^22^. This study found that AP can reverse cognitive impairment in aging mice, and improve their spatial learning and passive memory performance. BDNF is one of the most abundant neurotrophic factors in the central nervous system, playing a crucial role in nourishing and protecting nerves, as well as enhancing synaptic plasticity^23^. Synaptic related proteins, especially PSD95 and SYP, are markers of synaptic plasticity and play a crucial role in memory^24^. Research has confirmed that compared to 5-month-old mice, the levels of PSD95 and SYP proteins in 22-month-old mice are significantly downregulated^25^. This study found that the expression of BDNF, PSD95 and SYP in the brain tissue of aging mice decreased, further confirming the cognitive dysfunction of aging rats. AP intervention can effectively reverse the decline of BDNF, PSD95 and SYP during the aging process, indicating that AP improves cognitive dysfunction in aging rats by improving synaptic plasticity. One factor contributing to age-related cognitive decline is the increase in oxidative stress^26^. Previous studies have found that supplementing with concentrated apple juice can improve oxidative damage and cognitive decline in food deficient aging mice^21^. The research results of Zhonghua Liu et al. revealed that theaflavins can improve cognitive impairment in aging mice induced by D-galactose by increasing their antioxidant capacity^27^. Consistent with previous studies, our study found that AP maintained the redox balance in mice, exhibiting strong antioxidant effects, increasing CAT, SOD, T-AOC, GSH-px activity, and reducing MDA levels.

Most elderly people develop a mild pro-inflammatory state, which is associated with increased susceptibility to various age-related diseases, and pro-inflammatory cytokines IL-1β, IL-6 and TNF-α play a crucial role in the amplification cascade of inflammation^28^. Research reports that exposure to D-galactose can increase TNF-α, IFN- γ, IL-6 and IL-1β levels of pro-inflammatory factors^29^. In our study, aging mice showed an increase in serum pro-inflammatory cytokine levels, and AP administration for 3 months could reverse this phenomenon. TLR4/NF-κB is a classic inflammation related signaling pathway that plays an important role in mediating inflammation^30^. Activation of TLR4 leads to NF-κB enters the nucleus from the cytoplasm, leading to the release of a large number of pro-inflammatory factors^31^. Upregulation of the TLR4/NF-κB inflammatory pathway in the mouse intestine can be observed in the SAMP6 mouse model, leading to an inflammatory response in the mouse intestine^32^. Mao et al*.* revealed that D-galactose-induced aging mice exhibit activation of the TLR4/NF-κB signaling pathway in intestinal tissue, and downregulation of TLR4 and NF-κB protein expression can repair aging induced intestinal barrier damage^33^. In this study, we revealed that AP can downregulate the activation of the TLR4 signaling pathway in intestinal tissue, inhibit the release of pro-inflammatory factors, and reduce systemic and intestinal inflammatory responses in aging mice.

Chronic progressive inflammation and oxidative stress with age can disrupt the intestinal epithelial barrier and increase intestinal permeability^34^. Previous studies have found that in elderly rats, the presence of goblet cells producing neutral mucin is lower, which leads to a weaker mucus layer and lower buffering capacity^35^. D-galactose-induced mice exhibit increased intestinal permeability and higher levels of serum endotoxin were observed. The AB/PAS staining results intuitively demonstrated the protective effect of AP on the intestinal barrier. Occludin and ZO-1 were tight junction (TJ) proteins that play a crucial role in maintaining intestinal epithelial cell adhesion and barrier homeostasis^36^. Previous studies have shown that with the increase of age, the body may undergo a series of changes, especially in the integrity of the intestine^37^. Dawn M.E. Bowdish et al*.* found that the intestinal permeability and inflammatory level of aging mice increased^38^. Tran et al*.* indicated that compared to young baboons, the expression of ZO-1 and occludin in colon tissue of elderly baboons was reduced, and permeability was significantly increased^39^. Previous study has revealed that apple extract can inhibit TNF-α induced TJ dysfunction was associated with increased expression of TJ proteins ZO-1 and occludin^40^. Li et al*.* reported that apple polyphenols can increase the protein expression of ZO-1 and Occludin, upregulate the protein levels of MUC-2 and TTF3, improve the integrity of the intestinal barrier and the function of goblet cells^41^. Similarly, we observed disruption of the intestinal mucosal barrier in aging mice, and administration of AP could reverse this result.

It has been confirmed that p21^waf1^ (p21) is associated with important biological functions such as regulating cell cycle, DNA repair, and apoptosis, while P16^lnk4a^ (p16), as a regulator of cellular aging, can reduce the risk of age-related diseases^58^. Due to the accumulation of p16 and p21 in various tissues during normal aging processes in rodents and humans, they are used as aging markers^42^. Previous study has observed an increase in p21 and p16 protein expression in liver and kidney tissues in D-galactose induced aging mouse model^43^. Masternak et al*.* have shown that Dasatinib and Quercetin alleviate intestinal aging in mice by reducing the mRNA levels of p16 and p21 in intestinal tissue^44^. Similarly, in this study, we observed that AP can reduce the expression levels of p16 and p21 caused by aging. However, the increased expression of these aging markers can also be caused by active macrophages^45^. We further performed SA-β-Gal staining on mouse colon tissue, and the results showed that AP can inhibit β-galactosidase activity and delay intestinal aging. SA-β-Gal positive aging cells were observed in a 5-FU induced intestinal aging model^46^. Research reports that when intestinal progenitor cells transform into absorbing or secreting cells and migrate towards the villous basement, cell proliferation stops as differentiated cells leave the crypt^47^. In our study, we observed a decrease in the number of Ki67^+^progenitor cells in elderly mice compared to young mice, and AP treatment rescued these progenitor cells. Aging is closely related to apoptosis, the TUNEL staining and apoptosis protein expression showed that AP can inhibit apoptosis in intestinal tissue^48^. In summary, these results indicate that AP can regulate intestinal anti-inflammatory and antioxidant capacity, repair intestinal mucosal barrier, inhibit cell apoptosis, and delay intestinal aging in mice.

The gut microbiota changes with age, affecting gut barrier function and regulating cognitive abilities through the gut brain axis^49^. *Francesco Marotta* revealed that age-related changes in gut microbiota composition include a decrease in microbial diversity, a decrease in dominant species abundance, and an increase in sub dominant species abundance^50^. In this study, we observed differences in gut microbiota diversity in aging mice, characterized by a decrease in α-diversity and isolation of β-diversity. Additionally, we also observed differences in species abundance. The genus *Helicobacter* contains over 35 species. *Helicobacter pylori* is the most important factor in human health, and it is associated with gastritis, precancerous gastric atrophy, and intestinal metaplasia^51^. In aging mice, we observed a high relative abundance of *Helicobacter*, and AP significantly reduced the abundance of this genus in the feces of aging mice. *Bilopila* belongs to the *Proteobacteria* and is an opportunistic pathogen, *B. wadsworthia* synergizes with a high-fat diet to promote higher inflammatory responses, intestinal barrier dysfunction, and abnormal bile acid metabolism, leading to higher glucose metabolism abnormalities and liver fat production^52^. *Bilopila* can reproduce and accumulate in large quantities under the dual conditions of ketogenic diet and intermittent hypoxia, thereby causing damage to hippocampal function and increasing the risk of cognitive impairment^53^. Compared with young mice, *Bilopila* was significantly enriched in fecal bacteria of aging mice, and the administration of AP reversed this result.

Notably, the relative abundance of *Bacteroides* increased in aging mice after AP treatment (OA), and Lefse analysis found that *Bacteroides vulgatus* was enriched in the OA group. *Bacteroides vulgatus* is one of the main *Bacteroides* in the human gut, with various beneficial effects. Previous study has reported that *Bacteroides vulgatus* and *Bacteroides dorei* can reduce the production of lipopolysaccharide in intestinal microorganisms and inhibit atherosclerosis^54^. Meanwhile, *Bacteroides vulgatus* can also reduce the expression of colon TNF-α, IL-1β and IL-6 induced by DSS and improve colitis in mice^55^. In addition, we also observed an increase in the genus *Lactobacillus* in the OA group, including *Lactobacillus faecis* and *Lactobacillus taiwanensis*. Jie Yu et al*.* revealed that *Lactobacillus faecis* belongs to *Lactobacillus* with high SCFAs production^56^. Previous studies have shown that the abundance of *Lactobacillus taiwanensis* was low in colorectal cancer and cervical tumors, indicating that *Lactobacillus taiwanensis* may have a protective effect in the occurrence and development of colorectal and cervical tumors^57^. Yan Qing Li et al. reported that *Lactobacillus taiwanensis* has antibacterial activity and applications in regulating intestinal immunity^58^. *Parabacteroides goldsteinii* showed high relative abundance in the OA group. Studies have shown that *Parabacteroides goldsteinii* has shown good prebiotic effects, including relieving chronic obstructive pulmonary disease, improving obesity and type 2 diabetes, and treating autism^59^. Kristiansen et al. revealed a correlation between the levels of inflammatory cytokines and the abundance of specific bacterial taxa, including *Lachnospiraceae bacterium 28–4* and *Alistipes inops*^60^. We observed an increase in the relative abundance of *Alistipes inos* in aging mice, while its relative abundance decreased after AP administration. In addition, there were high relative abundances of *Parasutterella* and *Pantoea*, as well as *Burkholderiales bacterium YL45* and *Pantoea agglomerans* in the fecal microbiota of control group mice (YC). *Parasutterella* is known to be a high consumer of L-cysteine, and L-cysteine is known to improve the blood sugar level of rodents, so *Parasutterella* is considered to be important in the occurrence and development of type 2 diabetes and obesity^61^. *Pantoea* is a rare pathogen of human infectious diseases. *Pantoea agglomerans* is a genus of *Pantoea*, which may be the cause of opportunistic infections in humans, mainly from wounds made of plant materials or as hospital acquired infections, and occurring in individuals with weakened immune function^62^.

In summary, AP can improve the antioxidant capacity of aging mice, inhibit intestinal inflammation through the TLR4/NF-κB signaling pathway, repair the intestinal mucosal barrier, regulate intestinal microbiota, and ultimately alleviate intestinal aging and improve age-related cognitive dysfunction. Meanwhile, it was found that administering AP to young mice can reduce the relative abundance of opportunistic pathogens. These results indicate that daily intake of apples is safe and effective in improving intestinal aging during the aging process.

## Materials and methods

This study was carried out in compliance with the ARRIVE (Animal Research: Reporting of In Vivo Experiments) guidelines. All animal experiments complied with “the Guide for the Care and Use of Laboratory Animals”, and were approved by the Animal Experiment Ethics Committee of Nanchang University (NCULAE-20221031089). The methods of this study were informed to all the authors and were conducted in accordance with relevant guidelines and regulations.

### Animal model and treatments

Twelve male C57BL/6 J mice aged 6–8 weeks were obtained from Hunan Tianqin Experimental Animal Co., Ltd. (Hunan, China) and raised naturally until 18 months old. Additionally, purchase 12 male C57BL/6 J mice aged 6–8 weeks and adaptively feed them for one week before use in the experiment. Afterwards, the mice were randomly divided into four groups: Young mice group (YC group), aging mice group (OM group), aging mice with 100 mg/kg AP group (OA group), young mice with 100 mg/kg AP group (YA group). The entire treatment cycle lasts for 3 months. The AP in this study were commercially purchased from Sichuan Vicki Biotechnology Co., Ltd. (WKQ-0790583). All mice were subjected to the same temperature (22 ± 2 ℃), humidity (55–60%), 12 h/12 h of light/dark cycle, and free consumption of standard rodent feed and water.

### Morris water maze test

After 3 months of treatment, the spatial learning ability of all mice was evaluated in a water maze, consisting of black organic glass rectangular jars measuring 73, 42, and 20 cm in length, width, and depth, respectively. The container includes one starting point, one terminal platform, and four platform free facilities. Fill the water maze with a depth of 12 cm and control the temperature at 22 ± 1 ℃. On the first day of training, let each mouse stay on the terminal platform for 30 s to identify the location and place it in the water facing the pool wall. At the first starting point, include non-platform facilities and record the escape latency period for each experiment to find the terminal platform. The mouse cannot find the platform within 1 min, and the swimming time would be set at 1 min. The mice underwent the experiment continuously for 6 days. For all experiments, the effect of AP on delaying aging in mice was measured and calculated using a computerized video imaging analysis system^63^.

### H&E, AB/PAS, IHC, TUNEL and IF staining

Brain and colon tissue samples were fixed with 4% paraformaldehyde, embedded in paraffin, cut into 5–6 μm thick sections, dewaxed, dehydrated, and stained with hematoxylin eosin (H&E) histology according to the standard procedure. Immunohistochemical staining (IHC) was used to evaluate the expression of aging related proteins Ki67 (1:1000; Cat# 28,074-1-AP; Proteintech Group, Inc.) and p21 (1:200; Cat# 28,248-1-AP; Proteintech Group, Inc) in colon tissue. Colon tissue sections were subjected to ZO-1 (1:100; Cat# 21,773-1-AP; Proteintech Group, Inc.) and Occludin (1:200; Cat# 27,260-1-AP; Proteintech Group, Inc.) immunofluorescence (IF) staining to detect the expression of colon barrier proteins. Then we used Alishin blue/Periiodate Schiff staining (AB/PAS) to stain colon tissue sections to evaluate the number of goblet cells in the colon. The terminal deoxynucleotidyl transferase‑mediated dUTP nick end labeling (TUNEL) assay kit (Cat# T2196; Solarbio, Inc.) was used to evaluate the apoptosis of mouse colon tissue cells. Finally, images are collected using a microscope or fluorescence microscope (Olympus, Japan) and analyzed using ImageJ software (version 1.8.0).

### Nissl staining

Perform dewaxing and dehydration on the slices according to the above method^64^. Firstly, soak the slices in 100% ethanol for about 5 min, 95% ethanol for 30 s, and 70% ethanol for 30 s. Then wash three times with ultrapure water, soak in 1% toluidine blue for 60 min, and wash three times with ultrapure water. After washing the dye with distilled water, ethanol is dehydrated. Finally, soak the slices in xylene for 2 h and seal them with neutral glue.

### SA-β-Gal staining

Senescence-associated β-galactosidase (SA-β-Gal) staining of colon tissue was performed utilizing a SA-β-Gal staining kit (Cat# G1580; Solarbio, Inc.). Frozen colon tissue sections were fixed with 4% formaldehyde at room temperature for 10 min. Rinse the slide with PBS for 3 times and keep it for 5 min, and then incubate with freshly prepared SA-β-Gal staining solution overnight in a humidification chamber at 37 ℃. The tissue sections were washed twice in PBS for 10 min at room temperature and then further stained with eosin. Finally, the image was collected by optical microscope, and the expression intensity of SA-β-gal was analyzed by ImageJ software (version 1.8.0).

### Measurement of inflammatory cytokines and antioxidant indexes in serum

Mouse blood was centrifuged at 1000 g, 15 min, and 4 ℃ to obtain serum. Serum inflammatory factors were detected according to the kit provided by Camilo Biological Interleukin-1β (IL-1β, Cat#2 M-KMLJM211201m), IL-6 (Cat#2 M-KMLJM219451m), Tumor necrosis factor-α (TNF-α, Cat#2 M-KMLJM220051m), and the absorbance was measured at 450 nm by enzyme-labeled instrument. Oxidative stress biomarkers, total antioxidant capacity (T-AOC; Cat# A015-2-1), superoxide dismutase (SOD; Cat#A015-2-1), catalase (CAT; Cat#A007-1-1), glutathione peroxidase (GSH-px; Cat#A005-1-2), and malondialdehyde (MDA; Cat#A003-1-2) were determined according to the manufacturer's protocol.

### Western blotting analysis

Western blotting analysis was performed according to the previous description. In short, prepare colon tissue proteins using RIPA lysate buffer containing protease and phosphatase inhibitors. After standardizing the sample to the same protein concentration using the bicinchoninic acid protein assay kit (BCA) protein detection kit, analyze an equal amount of protein using 6% -12% SDS-PAGE and transfer it onto a polyvinylidene fluoride membranes (PVDF) membrane. Block the membrane with 5% skim milk (in Tris-buffered-saline-Tween-20 buffer) and incubate with the designated primary antibody BDNF (1:1000; Cat# 25,699-1-AP; Proteintech Group, Inc.); PSD95 (1:1000; Cat# 20,665-1-AP; Proteintech Group, Inc.); SYP (1:4000; Cat# 17,785-1-AP; Proteintech Group, Inc.); TLR4 (1:750; Cat# sc‑293,072; Santa Cruz Biotechnology, Inc.); NF-κB (p65; 1:2000; Cat# 10,745‑1‑AP; Proteintech Group, Inc.); phosphorylated (p)-NF-κB (pp65; 1:1000; Cat# 3033; Cell Signaling Technology, Inc.); Bax (1:2000; Cat# 50,599-2-Ig; Proteintech Group, Inc.); Bcl-2 (1:5000; Cat# 68,103-1-Ig; Proteintech Group, Inc.); Zona occludens protein 1 (ZO-1; 1:5000; Cat# 21,773-1-AP; Proteintech Group, Inc.); Occludin (1:2000; Cat# 27,260-1-AP; Proteintech Group, Inc.); glyceraldehyde-3-phosphate dehydrogenase (GAPDH; 1:5000; Cat# 60,004-1-Ig; Proteintech Group, Inc.) at 4℃ overnight, then incubate the membrane with the secondary antibody (1:5000, Proteintech Group, Inc.) at room temperature for 1 h. Finally, the protein expression was using enhanced chemiluminescence reagent (ECL).

### DNA extraction and 16S rDNA gene sequencing

Genomic DNA of mouse feces was extracted, and the V4 region of 16S ribosomal DNA (rDNA) gene was amplified by primers (515F, 5'-AYTGGGYDTAAAGNG-3'; 806R, 5'-TACNVGGGTATCTAATCC-3'), and sequenced by Illumina Novaseq platform. The obtained high-quality sequence takes 97% similarity clustering as the operation classification unit (OUT). According to the annotation of taxa, the relative abundance of total bacteria in each sample is divided into different taxonomic levels (phylum, class, order, family, genus and species). The data were analyzed using QIIME2 (version 2019.4) (GenBank accession number PRJNA1093364)^65^.

### Statistical analysis

GraphPad Prism software (version 9.0) was used to evaluate the statistical significance of the comparison between the experimental groups by one-way ANOVA with Tukey multiple comparison test. Data were considered significant at *P* < 0.05, with * *P* < 0.05, ** *P* < 0.01.

### Supplementary Information

Supplementary Information.

## Data Availability

Data is provided within the manuscript or supplementary information files.
