# Supplementary material for: Apple polysaccharide improves age-matched cognitive impairment and intestinal aging through microbiota-gut-brain axis
Source: Sci Rep. 2024 Jul 13;14:16215. doi: 10.1038/s41598-024-67132-4 (PMC11246462; doi:10.1038/s41598-024-67132-4)
Supplement: Supplementary file 1 — Supplementary Information. [file 41598_2024_67132_MOESM1_ESM.pdf]

Origin images of western blotting

Figure S1

YC OM OA YA

BDNF 28kDa

PSD95 95kDa

SYP 38kDa

GAPDH 36kDa

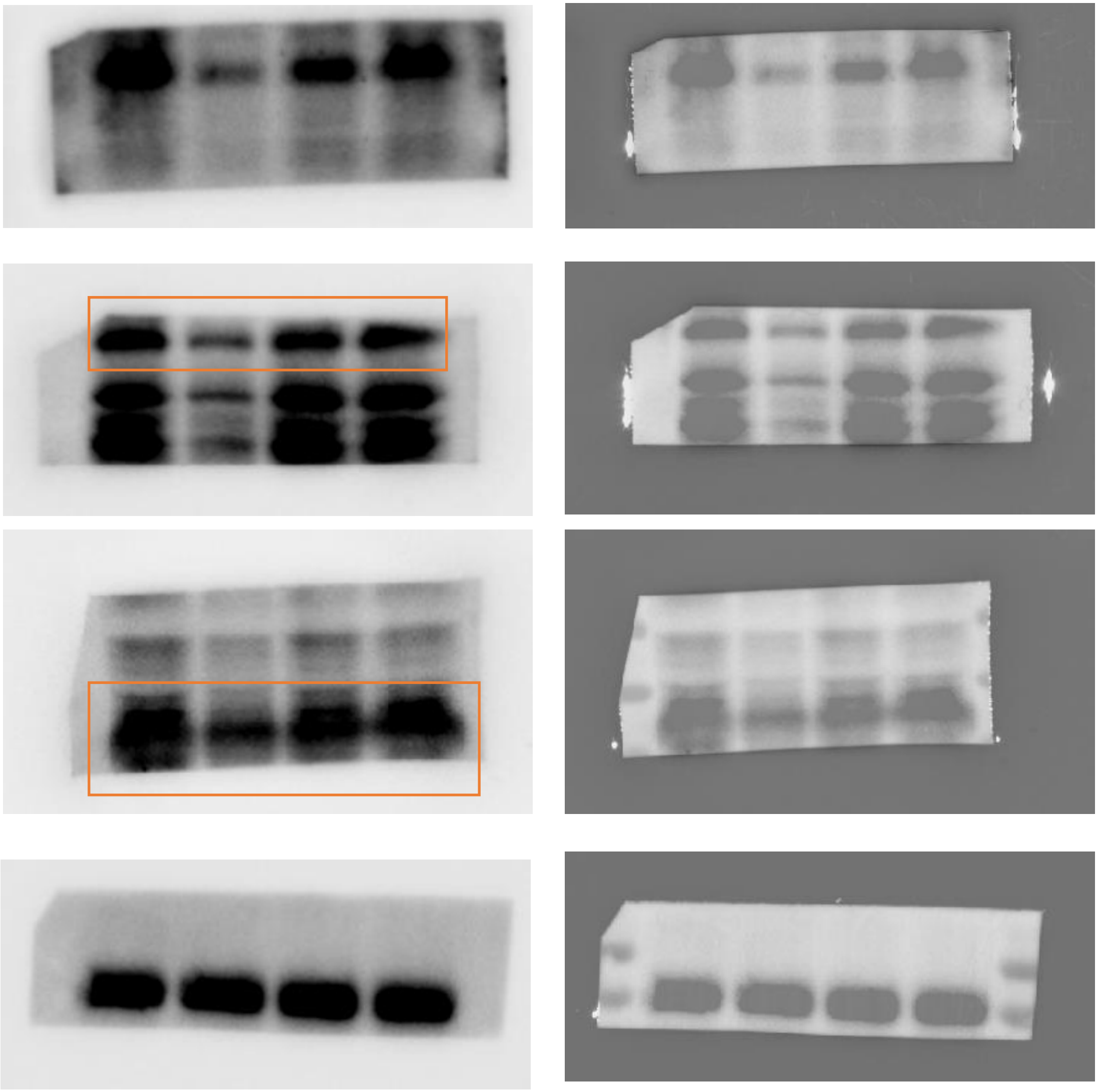

These membranes were cut prior to hybridization with antibodies

Figure S2

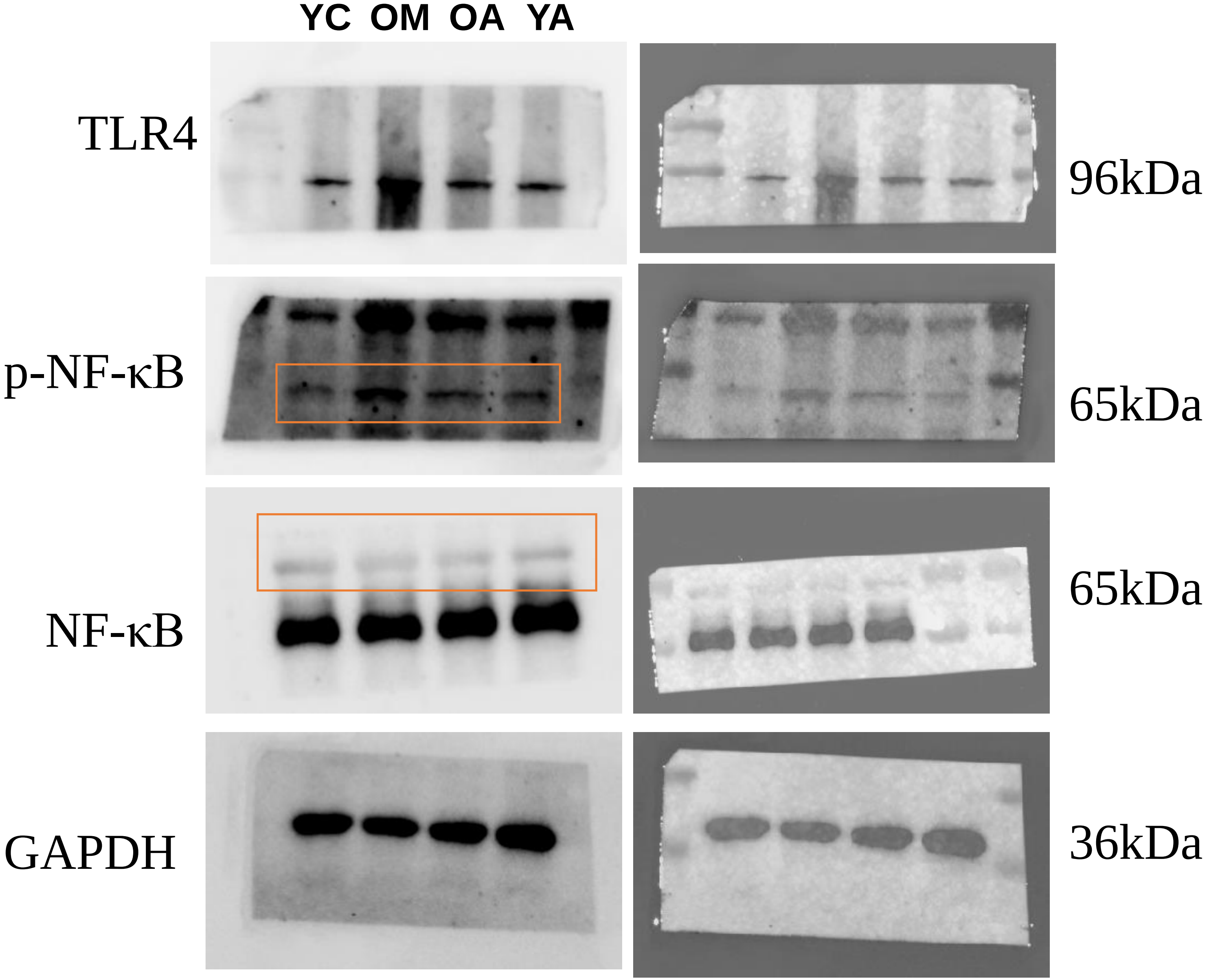

These membranes were cut prior to hybridization with antibodies

Figure S3

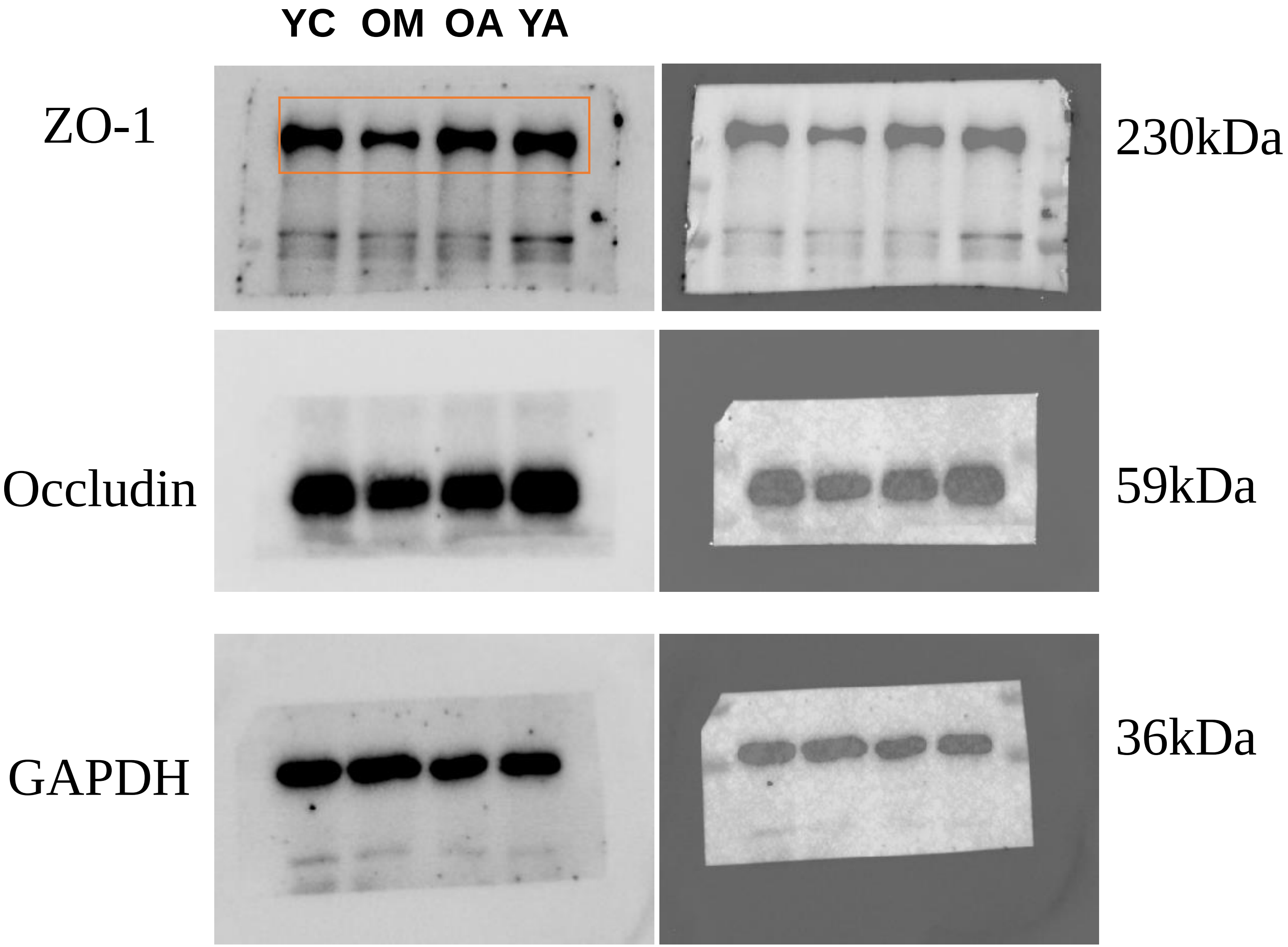

These membranes were cut prior to hybridization with antibodies

Figure S4

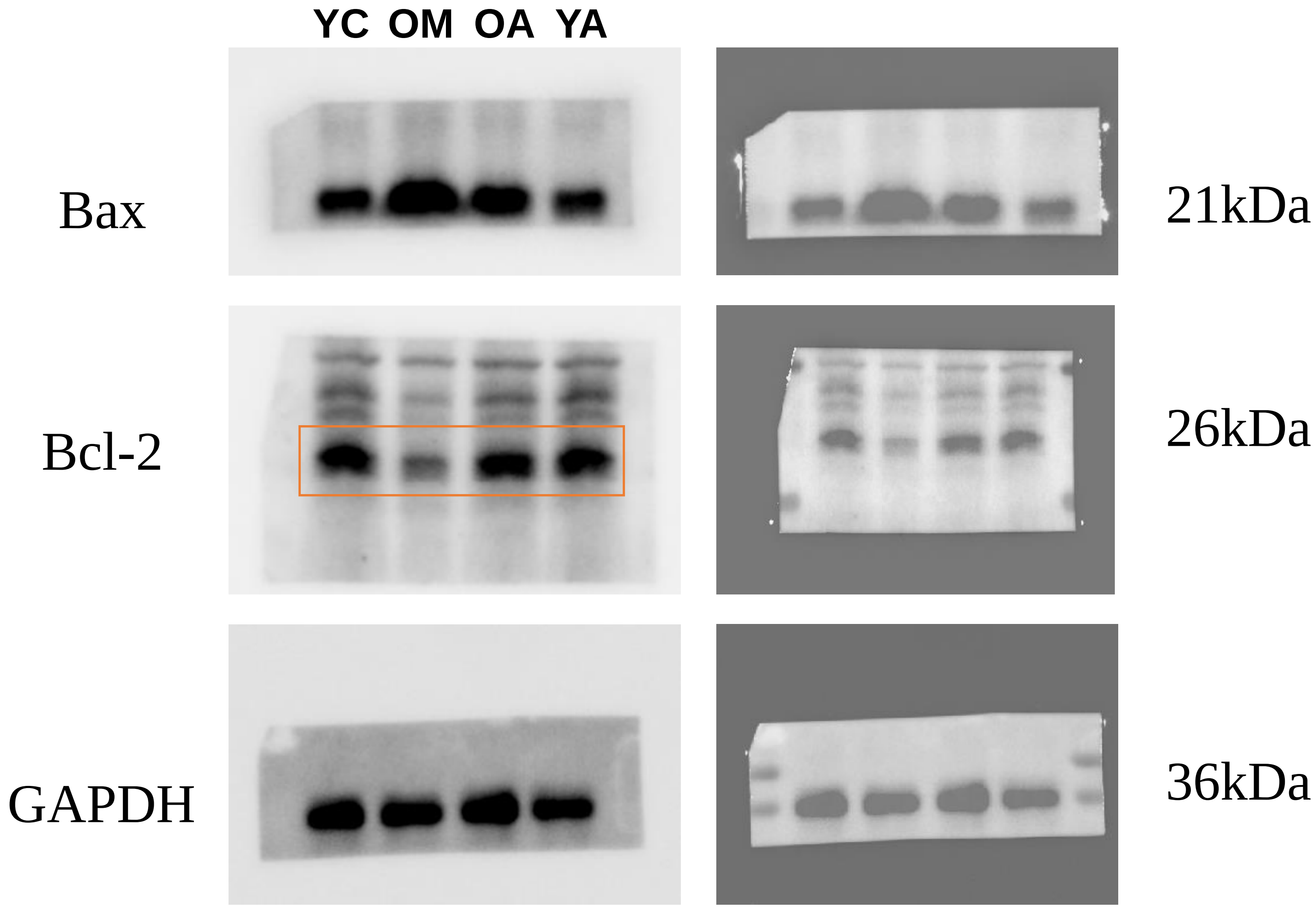

These membranes were cut prior to hybridization with antibodies
